# Gastrectomy with Roux-en-Y reconstruction as a lean model of bariatric surgery^☆^^☆☆^

**DOI:** 10.1016/j.soard.2018.01.039

**Published:** 2018-05

**Authors:** Geoffrey P. Roberts, Richard G. Kay, James Howard, Richard H. Hardwick, Frank Reimann, Fiona M. Gribble

**Affiliations:** a Metabolic Research Laboratories and MRC Metabolic Diseases Unit, Wellcome Trust MRC Institute of Metabolic Science, University of Cambridge, Cambridge, United Kingdom; b Cambridge Oesophago-gastric centre, Addenbrooke’s Hospital, Cambridge, United Kingdom; c LGC Limited, Newmarket Road, Fordham, Cambridgeshire, United Kingdom

**Keywords:** Bariatric surgery, Roux-en-Y gastric bypass, gastrectomy, glucagon, glucagon-like peptide-1 (GLP-1), hypoglycemia

## Abstract

**Background:**

Altered enteroendocrine hormone responses are widely believed to underlie the beneficial effects of bariatric surgery in type 2 diabetes. While elevated postprandial glucagon-like peptide-1 (GLP-1) is considered one of the mediators, increased postprandial glucagon levels have recently been implicated.

**Objectives:**

We investigated hormonal responses in lean patients after prophylactic total gastrectomy (PTG), as a model of Roux-en-Y gastric bypass without the confounding effects of obesity or massive weight loss.

**Setting:**

University hospital, United Kingdom.

**Methods:**

Ten participants after PTG and 9 healthy volunteers were recruited for oral glucose tolerance tests. Plasma glucose, insulin, GLP-1, peptide YY, glucose-dependent insulinotropic-polypeptide, glucagon, oxyntomodulin, glucagon_(1-61)_, and glicentin levels were assessed using immunoassays and/or mass spectrometry.

**Results:**

PTG participants exhibited accelerated plasma glucose appearance, followed, in 3 of 10 cases, by hypoglycemia (<3 mM glucose). Plasma GLP-1, peptide YY, glucose-dependent insulinotropic-polypeptide, glicentin, and oxyntomodulin responses were elevated, and glucagon appeared to rise in PTG participants when measured with a glucagon-specific enzyme-linked immunosorbent assay. We revisited the specificity of this assay, and demonstrated significant cross-reactivity with glicentin and oxyntomodulin at concentrations observed in PTG plasma. Reassessment of glucagon with the same assay using a modified protocol, and by liquid chromatography-mass spectrometry, demonstrated suppression of glucagon secretion after oral glucose tolerance tests in both PTG and control cohorts.

**Conclusions:**

Care should be taken when assessing glucagon levels in the presence of elevated plasma levels of other proglucagon products. Substantial elevation of GLP-1 and insulin responses after PTG likely contribute to the observed hypoglycemia, and mirror similar hormone levels and complications observed in bariatric weight loss patients.

Altered glucagon-like peptide-1 (GLP-1) secretion is a well-established mediator of improved glucose homeostasis after bariatric surgery, but controversy exists over the relative importance of other proglucagon-derived peptides [1]. The glucagon gene encodes a 180-amino-acid preprohormone, which, after removal of a 20-amino-acid signal peptide, is differentially cleaved to glicentin_(1-69)_, oxyntomodulin_(33-69)_, glucagon_(33-61)_, GLP-1_(78-108)_, glucagon like peptide-2 (GLP-2_(126-158)_), glicentin-related peptide_(1-30)_, and the major proglucagon fragment_(72-158)_. There also exists evidence for the production of an N-terminal peptide called glucagon_(1-61)_ that includes the sequences of glicentin-related peptide_(1-30)_ and glucagon_(33-61)_ [2], [3]. In this article, the unqualified nomenclature glucagon refers to glucagon_(33-61)_. It is generally accepted that intestinal L-cells express prohormone convertase PC1/3 and preferentially process proglucagon to glicentin, oxyntomodulin, GLP-1, and GLP-2, while pancreatic α-cells express PC2 and preferentially produce glucagon, glicentin-related peptide, and the major proglucagon fragment [4].

The sequence of glucagon_(33-61)_ is nested within that of both oxyntomodulin and glicentin and shares a common N-terminus with oxyntomodulin, explaining historical challenges with analysis of pancreatic glucagon concentrations in plasma. A study using immunoassays and mass spectrometry to analyze the plasma of patients after total pancreatectomy found evidence of postprandial elevation of glucagon_(33-61)_, suggesting it may also have a nonpancreatic source [5]. Elevated postprandial glucagon levels were also recently reported in patients who had undergone Roux-en-Y gastric bypass (RYGB) surgery [6], [7], leading the authors to suggest that aberrant proglucagon processing in the gut might produce glucagon_(33-61)_ after bariatric surgery.

Here, we describe the plasma profiles of proglucagon-derived and other gut peptides in healthy volunteers and patients after prophylactic total gastrectomy (PTG) to prevent familial gastric cancer. Our lean patients are of particular interest, in that the reconstruction, apart from the total resection of the stomach, is similar to conventional RYGB surgery used to treat morbid obesity and results in similar rerouting of nutrients to the jejunum without delay after ingestion. The operative effects can thus be described without the confounding effect of presurgical morbid obesity. This makes gastrectomy with Roux-en-Y an interesting model of the metabolic effects of gastric bypass [8].

Using standard and high-specificity immunoassays and mass spectrometry, we observed elevated GLP-1, peptide YY (PYY), and insulin levels after glucose ingestion in PTG patients, with consequent hypoglycemia in several participants. Postprandial secretion of pancreatic-type glucagon_(33-61)_ was not enhanced after PTG, and assay cross reactivity rather than gut-derived glucagon_(33-61)_ is the likely explanation for apparently raised glucagon levels observed after glucose tolerance testing in this patient group.

## Methods

Ten patients who had previously undergone PTG and 9 matched healthy volunteers were recruited for oral glucose tolerance tests (OGTTs). The study was approved by the local National Health Service Research Ethics Committee and conducted in accordance with the ethical standards of the Helsinki Declaration of 1975.

### Surgery

**Fig. 1:**
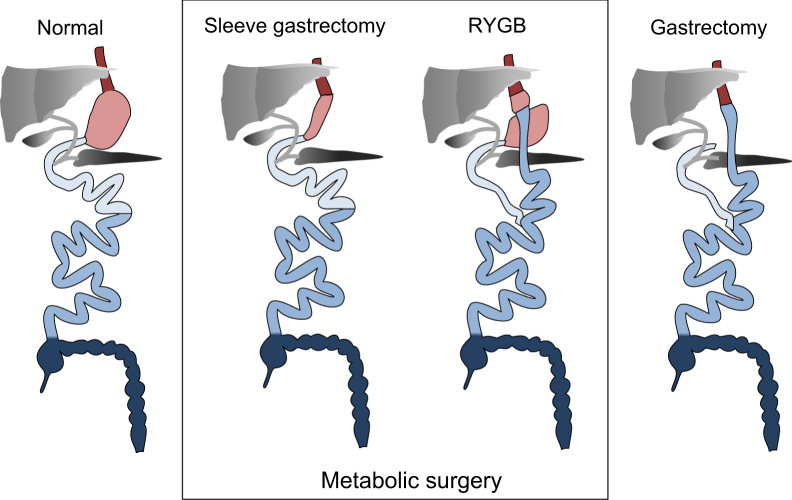
Postoperative intestinal anatomy. Schematic representation of gastrointestinal anatomy before surgery and after sleeve gastrectomy, Roux-en-Y gastric bypass, or total gastrectomy with Roux-en-Y reconstruction.

All gastrectomy participants underwent a D1 prophylactic total gastrectomy with standard Roux-en-Y reconstruction (50-cm alimentary and biliopancreatic limbs) and were>6 months postsurgery at the time of study (Fig. 1).

### Glucose tolerance test

After an overnight fast, all participants drank 50 g of glucose in 200 mL water within a 5-minute period. A 50-g OGTT was selected as it elicits a significant gut hormone response in postgastrectomy patients and because of patient concern regarding adverse effects with a greater glucose load. Blood samples were collected immediately before glucose ingestion and at 15, 30, 45, 60, 90, 120, 150, and 180 minutes postingestion.

### Plasma samples

Blood was collected into ethylenediaminetetraacetic acid tubes (mass spectrometry for oxyntomodulin), lithium heparin tubes (insulin immunoassay, glucose, mass spectrometry for glucagon), and ethylenediaminetetraacetic acid tubes treated with DPP4 inhibitor and aprotinin (all other immunoassays). Samples were immediately centrifuged for 10 minutes at 3500 g at 4°C. Plasma aliquots were snap frozen on dry ice and stored at −80°C within 30 minutes of phlebotomy. Total GLP-1 was measured using Mesoscale Discovery (MD, USA) and Mercodia (Uppsala, Sweden) assays. Total GIP and total PYY were measured using Mesoscale Discovery assays. Glucagon was measured using the Mercodia sandwich immunoassay kit following the manufacturer’s standard kit protocol (referred to here as “standard” glucagon). After discussion with Mercodia, some samples were reanalyzed using a modified assay protocol designed to reduce cross reactivity with other proglucagon species (referred to as “specific” glucagon; for protocols see supporting material; n = 10 PTG, n = 8 control, time points 0 and 30 min). Glicentin and glucagon_(1-61)_ were assayed using Mercodia sandwich immunoassays, and oxyntomodulin by mass spectrometry peptidomics after extraction involving acetonitrile precipitation as previously described [9]. Glucagon was also measured by liquid chromatography-mass spectrometry (LC-MS, n = 10 gastrectomy and n = 7 controls, as insufficient plasma was available to repeat the assay for 1 control participant) using a method based on that previously described [10]. Specifically, the protocol was modified to account for the expected concentration of glucagon and volume of plasma available and to use depleted plasma, rather than buffer, as an improved calibration matrix. Samples with measured LC-MS glucagon below the level of detection (4.3 pM) were assigned a value of 4.3 pM. Cross reactivity of the standard and specific glucagon assays to glicentin and oxyntomodulin were measured by running multiple concentrations of glicentin and oxyntomodulin standards through the glucagon assays. Cross reactivity is reported as 100 × glucagon assay read / concentration of peptide (pM).

Data were collated in Microsoft Excel (Redmond, WA) and analyzed using R-studio and *ggplot2*. Results are presented as mean ± standard error.

## Results

### Participant characteristics

Control and gastrectomy groups were closely matched for sex, age, and waist:hip ratio, but the gastrectomy group had a slightly lower body mass index. All gastrectomy participants were carriers of a pathogenic mutation in *CDH1* and had undergone total gastrectomy as a prophylactic measure before the development of invasive cancer. Participants were studied after achieving stable postoperative weight, and not earlier than 6 months postoperatively. All participants had a glycosylated hemoglobin level within the nondiabetic range, and none were taking antidiabetic medication (Table 1).

**Table 1:** Participant demographic characteristics

Variable (mean, standard deviation)	Gastrectomy (n = 10)	Control (n = 9)
BMI, kg/m^2^	22.0 (2.3)	25.5 (3.9)
Age, yr	34.9 (7.5)	29.9 (7.2)
Waist:hip ratio	0.85 (0.07)	0.86 (0.12)
Sex (M:F)	7:3	6:3
HbA1C	36.2 (2.1)	32.2 (2.7)

BMI = body mass index; M = male; F = female; HbA1C = glycated hemoglobin.

### 3-hour glucose, insulin, GLP-1, PYY, GIP, and glucagon (standard assay) profiles

**Fig. 2:**
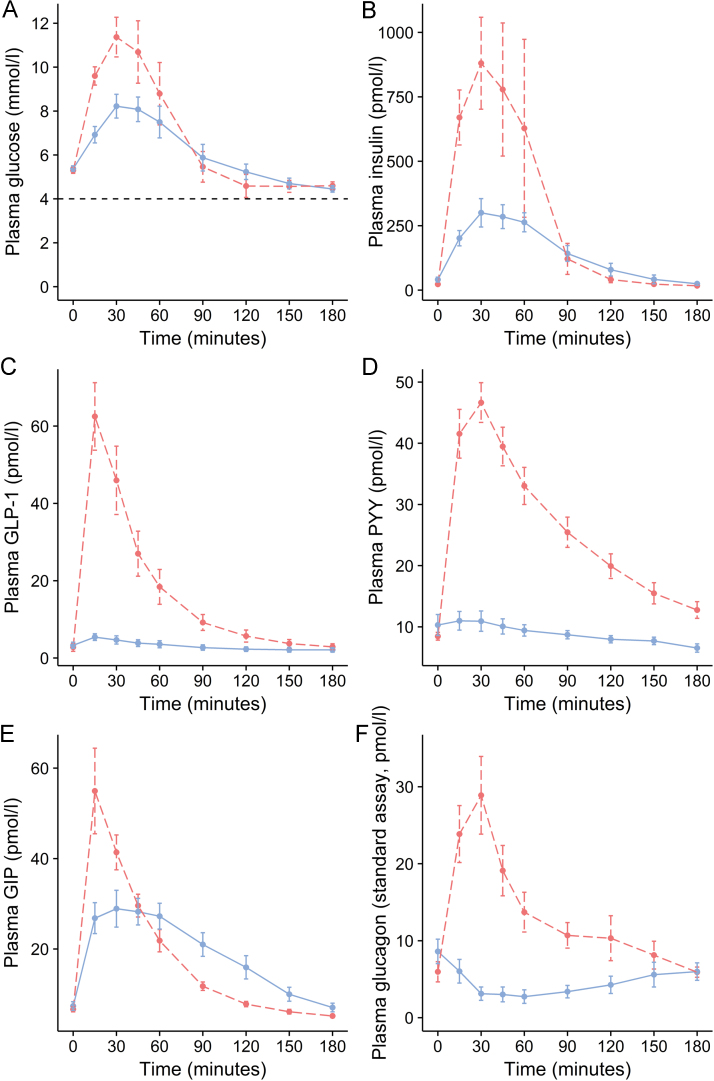
Oral glucose tolerance test results. (a–f) Plasma glucose, insulin, glucagon-like peptide-1, peptide YY, glucose-dependent insulinotropic peptide, and “standard” glucagon concentrations after oral glucose tolerance test in 10 postgastrectomy participants (red/dashed) and 9 healthy controls (blue/solid). All values are mean ± standard error.

After a 50-g OGTT, plasma glucose concentrations rose and fell faster in the gastrectomy group, with 3 patients exhibiting hypoglycemia (plasma glucose<3 mM). Insulin concentrations were higher in gastrectomy compared with control patients. Peak plasma GLP-1 levels were approximately 10-fold higher in the gastrectomy than control group. GLP-1 measured using the Mercodia and Mesoscale Discovery assays were closely matched. Peak plasma PYY levels were approximately 5-fold higher in the gastrectomy than control group and remained significantly elevated at the 180-minute time point. Peak plasma GIP levels were approximately 2-fold higher in the gastrectomy than control group, but were lower than control values from 60 minutes onward. Plasma “standard” glucagon concentrations fell after the OGTT in control patients but rose approximately 5-fold in the gastrectomy group between 0 and 30 minutes, after a similar temporal pattern to GLP-1, PYY, and GIP (Fig. 2).

### Proglucagon peptide concentrations

**Fig. 3:**
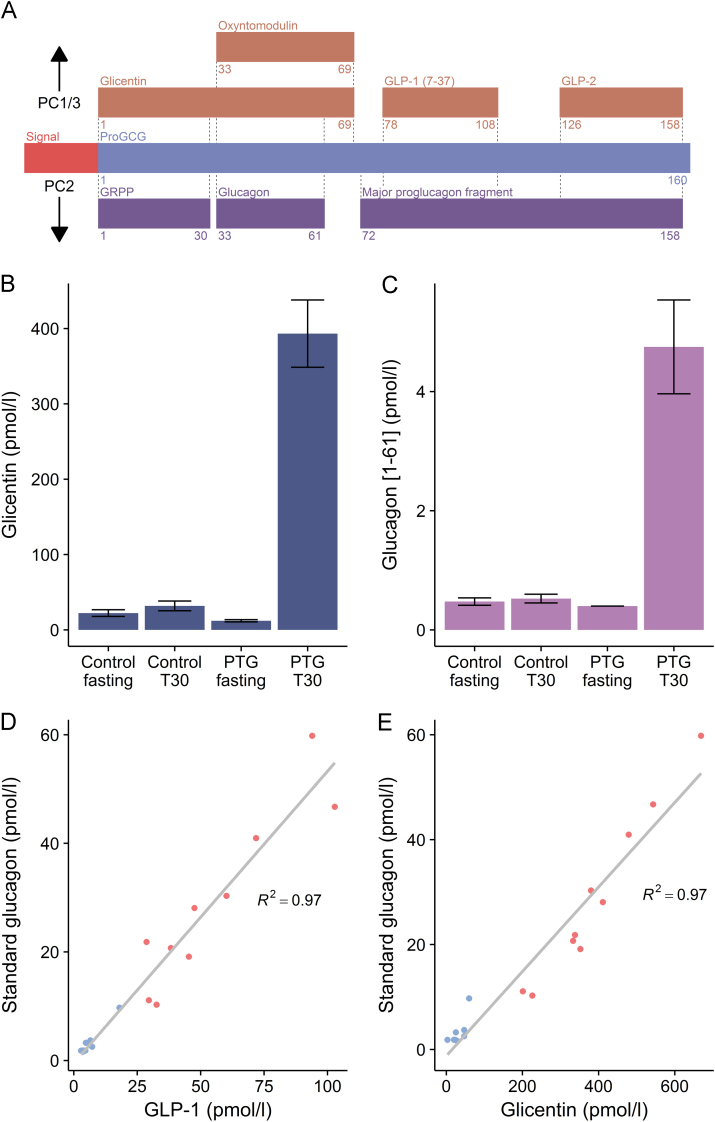
Proglucagon-derived peptide secretion. (a) Schematic of posttranslational products of the glucagon gene. (b, c) Plasma glicentin and glucagon_(1-61)_ concentrations at 0 and 30 minutes after oral glucose tolerance test (mean ± standard error). (d, e) Correlation between 30-minute plasma glucagon and glucagon-like peptide-1 (d) and glicentin (e) concentrations (blue control, red prophylactic total gastrectomy).

Plasma oxyntomodulin concentrations were below the lower detection limit in all fasting samples and in all but 1 sample at 30 minutes in the control group, but averaged approximately 46 ± 10 pM at 30 minutes in the gastrectomy group. Mean fasting glicentin concentrations were low in both groups but were markedly elevated in gastrectomy patients after OGTT, reaching approximately 400 pM at 30 minutes (*P*<.001 versus controls). Plasma concentrations of glucagon_(1-61)_ were below the detection limit in most fasting samples and averaged approximately 5 pM at 30 minutes in the gastrectomy group. Plasma GLP-1 and “standard” glucagon concentrations were highly correlated at the 30-minute time point, an unexpected finding as GLP-1 has been described to inhibit pancreatic glucagon secretion [11]. Plasma glicentin concentrations were also highly correlated with measured “standard” glucagon concentrations at the 30-minute time points, which suggested that the highly elevated glicentin concentrations at this time may have contributed to the “standard” glucagon assay result (Fig. 3).

### Glucagon assay cross-reactivity

Using the “standard” glucagon assay protocol, measured cross reactivity for glicentin and oxyntomodulin was low at concentrations typically found in control patients but was approximately 10% at the high concentrations found in the gastrectomy group. No relevant cross reactivity was observed using the “specific” glucagon assay protocol (Table 2).

**Table 2:** Cross reactivity of standard and specific glucagon assay protocols to measured concentrations of oxyntomodulin (OXM) and glicentin (GLN)

Peptide	Peptide concentration, pM	Standard glucagon assay cross-reactivity, %	Specific glucagon assay cross reactivity, %
OXM	226	11	1.7
OXM	22.6	6.6	Not measurable
GLN	295	8.9	Not measurable
GLN	99	3.8	Not measurable
GLN	29.5	Not measurable	Not measurable

### Modified glucagon assay and LC-MS results

**Fig. 4:**
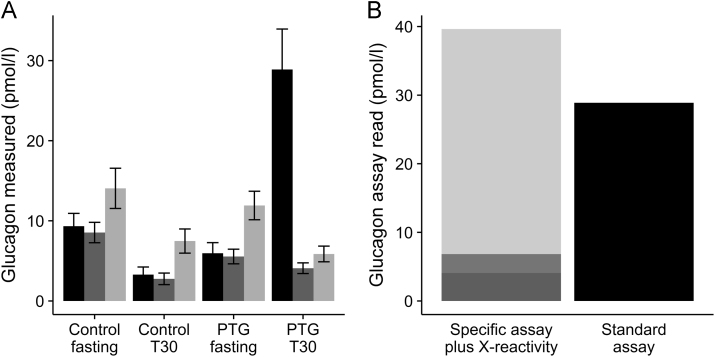
Glucagon results with liquid chromotography/mass spectrometry and high specificity assay protocol. (a) Glucagon measured using “standard” protocol (black), “specific” protocol (dark gray) and liquid chromotography/mass spectrometry (light gray), fasting and 30 minutes after an oral glucose tolerance test results in controls and PTG patients (mean ± standard error). (b) Thirty-minute plasma glucagon results using standard assay (black/right column) and summed “specific” glucagon concentration (dark gray) and predicted cross reactivity based on measured oxyntomodulin (middle gray) and glicentin (light gray). All values are the mean of prophylactic total gastrectomy participants.

Analysis of 0- and 30-minute samples using a modified glucagon immunoassay protocol (the “specific” assay) and separately LC-MS revealed that glucagon concentrations fell at 30 minutes in both gastrectomy and control patients, contradicting the “standard” glucagon assay result. Comparison of the “standard” glucagon assay result and the sum of the “specific” glucagon assay result and predicted glicentin and oxyntomodulin cross reactivity demonstrated that the initially described rise in plasma glucagon at the 30-minute time point in the gastrectomy group can be entirely explained by assay cross reactivity, particularly to the highly elevated glicentin concentrations (Fig. 4).

## Discussion

Mirroring findings in bariatric patients, lean postgastrectomy patients had high peak concentrations of GLP-1 and PYY after an OGTT, likely reflecting the rapid delivery of glucose to the distal small intestine where GLP-1– and PYY-producing L-cells are found at higher density [12]. Plasma insulin concentrations were higher after the OGTT in the gastrectomy group, and 3 of the patients developed hypoglycemia. Gastrectomy with Roux-en-Y reconstruction thus represents an opportunity to study the metabolic effects of gastric bypass surgery in a cohort without preexisting obesity or diabetes.

Consistent with the known co-production by L-cells of glicentin and oxyntomodulin, we also observed high levels of these peptides in gastrectomy patients 30 minutes after an OGTT. We were more surprised that glucagon concentrations were also elevated in the same samples, although similar high postprandial glucagon levels have been reported in patients after RYGB or pancreatectomy [5], [6], [7]. However, our results show that in the presence of high concentrations of glicentin and oxyntomodulin, assay cross reactivity was increased and sufficient to account for the apparently raised glucagon concentrations in these samples. The majority of the cross reactivity (92%) was attributable to glicentin, with a lesser contribution from the elevated oxyntomodulin concentration. We also cannot exclude the possibility that glicentin cross reactivity might contribute to the measured concentrations of glucagon_(1-61)_, as these peptides share the glucagon_(1-61)_ sequence.

Rerunning the glucagon assay with a revised protocol that included additional washing steps resulted in drastically lower measured “specific” glucagon concentrations in the post-OGTT samples of the gastrectomy group, without substantially lowering measured glucagon levels in the fasting samples or post-OGTT samples in the control group. Using this specific version of the assay, glucagon levels were found to fall in both control and gastrectomy patients 30 minutes after an OGTT. Analysis of the same samples by LC-MS confirmed the accuracy of the “specific,” not “standard,” assay results post OGTT in the gastrectomy group. These results suggest that the “specific” glucagon assay more accurately measures circulating pancreatic glucagon_(33-61)_ after an OGTT in patients with high glicentin and oxyntomodulin concentrations and that secretion of pancreatic glucagon is suppressed by glucose in gastrectomy and control patients.

Contrasting with the effects of ingesting pure glucose, in a study by Miyachi et al. [13], plasma glucagon concentrations measured by immunoassay and LC-MS were increased by approximately 25% in healthy patients 30 minutes after a mixed meal, which is likely attributable to other meal-derived components such as amino acids that stimulate pancreatic alpha cells directly. In bariatric patients after RYGB, glucagon concentrations measured using the standard Mercodia assay increased approximately 5-fold after a mixed meal and mirrored the concentrations reached after glucose ingestion in our PTG cohort [6]. Although a modest direct stimulation of alpha cells might have been triggered by amino acids derived from the test meal in this study of bariatric patients, our results suggest the high apparent glucagon concentrations might also be confounded by assay cross reactivity.

## Conclusions

The study of gut hormones has historically been fraught with analytic challenges, particularly in the measurement of peptides arising from the proglucagon peptide. Our data suggest that caution should be exercised in the interpretation of glucagon results performed using standard assay protocols in patients after gastrectomy or RYGB, in whom glicentin and oxyntomodulin concentrations are particularly elevated. Future studies aiming to measure plasma glucagon concentrations in circumstances of high glicentin and oxyntomodulin concentrations using the Mercodia assay should use the modified, high-specificity assay protocol available from the manufacturer. Our data do not support the idea that the gut secretes glucagon_(33-61)_ after bypass surgery.

## Disclosures

*FG is a consultant for Kallyope.*

## Appendix

### Supplementary data

Supplementary material
