# Supplementary material for: Gastrectomy with Roux-en-Y reconstruction as a lean model of bariatric surgery
Source: Surg Obes Relat Dis. 2018 May;14(5):562–8. doi: 10.1016/j.soard.2018.01.039 (PMC6191023; doi:10.1016/j.soard.2018.01.039)
Supplement: Supplementary file 1 — Supplementary material [file mmc1.docx]

**Supplementary data 1 – Glucagon assay methodology**

***Standard Mercodia glucagon assay protocol***

1. Prepare microplate wells for the number of samples and calibrators
2. Add 100µl Conjugate buffer to 25µl of sample or calibrator
3. Incubate on a plate shaker (700-900rpm) overnight (18-22hrs) at 2-8^o^C
4. Wash 6x with 700µl/well Wash buffer 1x solution
5. Add 200µl Substrate TMB to each well
6. Incubate for 15 minutes at room temperature
7. Add 50µl Stop solution to each well
8. Read optical density at 450nm within 30 minutes

***Modified / high specificity glucagon assay protocol***

1. Prepare microplate wells for the number of samples and calibrators
2. Add 100µl Conjugate buffer to 25µl of sample or calibrator
3. Incubate on a plate shaker (700-900rpm) overnight (18-22hrs) at 2-8^o^C
4. Wash 6x with 700µl/well Wash buffer 1x solution
5. Add 200µl Conjugate buffer to each well
6. Incubate on a plate shaker (700-900rpm) for 1 hour at 2-8^o^C
7. Wash 6x with 700µl/well Wash buffer 1x solution
8. Add 200µl Substrate TMB to each well
9. Incubate for 15 minutes at room temperature
10. Add 50µl Stop solution to each well
11. Read optical density at 450nm within 30 minutes
